# The influence of musical parameters and subjective musical ratings on perceptions of culture

**DOI:** 10.1038/s41598-023-45805-w

**Published:** 2023-11-24

**Authors:** John Melvin Treider, Jonas R. Kunst, Jonna K. Vuoskoski

**Affiliations:** 1 Department of Psychology, University of Oslo, Postboks 1094, Blindern, 0317 Oslo, Norway; 2 Department of Musicology, University of Oslo, Oslo, Norway; 3 RITMO Center for Interdisciplinary Studies in Time, Rhythm and Motion, Oslo, Norway

**Keywords:** Neuroscience, Psychology

## Abstract

Recent research suggests that music can affect evaluations of other groups and cultures. However, little is known about the objective and subjective musical parameters that influence these evaluations. We aimed to fill this gap through two studies. Study 1 collected responses from 52 American participants who listened to 30 folk-song melodies from different parts of the world. Linear mixed-effects models tested the influence of objective and subjective musical parameters of these melodies on evaluations of the cultures from which they originated. Musical parameters consistently predicted cultural evaluations. The most prominent musical parameter was musical velocity, a measure of number of pitch onsets, predicting more cultural warmth, competence and evolvedness and less cultural threat. Next, with a sample of 212 American participants, Study 2 used a within-subjects experiment to alter the tempo and dissonance for a subset of six melody excerpts from Study 1, testing for causal effects. Linear mixed-effects models revealed that both dissonance and slow tempo predicted more negative cultural evaluations. Together, both studies demonstrate how musical parameters can influence cultural perceptions. Avenues for future research are discussed.

## Introduction

Humans make social evaluations on a daily bases, based on a complex interplay of information from perceived external sources and pre-existing internal conceptions of others. Although a vast body of research has studied cultural perceptions in various domains, such as threat and likability^1^, few studies have investigated whether musical stimuli are able to modulate such ratings, or which musical parameters might inform listeners’ cultural evaluations. Thus, it is here the present study aimed to make a contribution and hopefully adjoin in the debate on whether music can influence relations *between* members from different groups and cultures.

Even though cultural biases tend to be relatively stable over time, Vuoskoski et al.^2^ found that participants who listened to either an Indian or a West African popular song prior to an implicit association test showed less bias against the respective culture. However, an earlier study indicated that music that sounded unconventional to listeners predicted more negative outgroup attitudes^3^. Thus, music may be able to both increase or reduce prejudice. This raises the question regarding which communicative aspects inherent in melodies may influence intergroup relations.

Music has been shown to effectively convey social intentions and functions as well as affective information reliably within a specific culture (i.e., Western music and Western listeners)^4,5^ and above chance level across different cultures^6–8^. Aucouturier and Canonne^9^ found that Western listeners were able to infer social intentions through acoustical cues of temporal and harmonic coordination that emerged from Western musicians’ dynamic interaction. Furthermore, Mehr et al.^10^ conducted a cross-cultural study that exposed participants from different parts of the world to song excerpts (without musical instruments) from 86 small-scale societies. Despite participants being unfamiliar with the cultures, the results indicated reliable cross-cultural inferences about the songs’ function (especially for dance and lullaby songs). Convergent evidence was provided by Balkwill et al.^7^, who demonstrated that certain musical cues for recognition (loudness, perceived complexity and tempo) predicted Japanese listeners’ perceptions of intended emotions of joy, sadness and anger. Remarkably, these patterns were observed for Japanese, Western and even Hindustani music, despite the latter culture’s music being unfamiliar to them. Taken together, these findings demonstrate that music can influence intercultural perceptions and attitudes even in the absence of understandable lyrical content.

Based on this existing research, it seems reasonable to assume that music has the ability to change people’s evaluation of others, for better or worse, and that these evaluations may be informed by perceived intentions conveyed by music. The influence of these perceived intentions, in turn, depends on several acoustical cues^5,11^. Based on the existing literature on these acoustical cues, we attempt to make inferences of their potential for modulating cultural evaluations. In the present research, we refer to such acoustical cues as musical parameters.

### The present research

Through two studies, the current research aims to shed light on how music and its specific parameters can influence people’s evaluation of cultural groups. Such an investigation has important implications, both practically and theoretically. Practically, considering the potential for music to increase contact and foster bonds between people, this research may provide important information about how music can be used to improve intercultural relations. Theoretically, investigating the specific musical parameters that exert an influence may advance theories of how music influences perceived social and emotional intentions, thereby shaping broader social evaluations.

In each study, we tested the effects of musical parameters on intercultural perceptions. In terms of musical parameters, we correlationally focused on originality, interval size, modality, general pitch variation and melodic velocity in Study 1, and experimentally on tempo and dissonance in Study 2. In terms of dependent outcomes, in both studies, we focused on basic dimensions of intergroup evaluation that have received attention in previous literature. First, we focused on the perceived warmth and competence of other cultures, which are the two main dimensions in the stereotype content model^12^. Second, based on integrated threat theory^13^, which posits that prejudice is a result of threat perceptions, we assessed the extent to which cultures were perceived as threatening or not. Finally, following the literature on dehumanization^14,15^, we assessed how “evolved” other cultures were perceived to be based in their music.

We acknowledge that by ensuring high experimental control using simple flute melodies and recruiting a uniform participant pool (White Americans), the present research has also divested the music and its recipients of any cultural context that one would normally be exposed to when perceiving a piece of music. The musical parameters under investigation are also only a small subset of possible parameters, and are based on the analysis and manipulation of simple melodies. It should also be noted that melody alone forms only a small part of a full musical experience, typically embedded in a cultural context. The ratings in the present research may thus primarily reflect mainstream North Americans’ culturally learned musical preferences, aesthetic norms and pre-existing biases of other cultures. We revisit these concerns in the general discussion of this paper.

## Study 1

The present study attempted to investigate whether objective musical parameters predict participants’ subjective musical ratings and cultural evaluations. Objective musical parameters and subjective ratings were obtained for short folk music melodies presented through an online survey and analysed using linear mixed models. The study is partly exploratory and partly tests a set of predictions. We expected that musical parameters previously associated with preference and perceived happiness, namely, originality and interval size^16,17^, would be related to positive ratings of music and evaluations of cultures. One way music’s originality can be operationalized is through the novelty or unusualness of its melody. Simonton^16^ created an algorithm based on tone-transition probabilities of musical themes by famous composers. He found that the popularity of a musical theme was positively associated with the originality of the theme. Moreover, Quinto et al.^17^ found that for Western listeners, perceived happiness was associated with larger average interval size in speech and melodies compared to perceived sadness and fear. The mode of music (e.g., the major and minor modes in Western music), is rooted in interval structures^18^. There is clear evidence that mode is related to emotional connotations in Western listeners, with the major mode being perceived as happy and the minor mode being perceived as sad^19–21^. Nevertheless, sad music is not necessarily disliked or deemed unpleasant, as preferences depend on a number of factors, such as personality, mood and musical training^22–24^). We therefore made no predictions regarding whether modality would affect musical and cultural ratings. Two of the parameters, melodic velocity and general pitch variation, have not, to the best of our knowledge, been investigated in association to preference or emotional connotations in music in the scientific literature, so no specific predictions were made with reference to these parameters.

### Methods

#### Participants

The participants were recruited via Amazon Mechanical Turk in August 2019. High quality responses were ensured by choosing participants with approval rates above 98 percent. The target sample size was set to a minimum of 50 participants. This sample size was chosen based on a rule of thumb by Hox^25^ for multilevel designs and a large-scale simulation study by Arend and Schäfer^26^. A total of 72 participants were thus initially recruited. Of these participants, 52 participants (17 female, 35 male, age range = 25–72, *M*_*age*_ = 41.33*, SD*_*age*_ = 12.24) fulfilled the inclusion criteria as they identified as White/Caucasian American. This selection was conducted to minimize cultural variation and prevent the possibility that some participants may belong to the cultures from which some of the music examples originated. The study lasted approximately 40 min, and participants were awarded $7 for participation.

#### Musical stimuli

First, melodies were collected from various MIDI databases: The Essen Folk Song Collection^27^, The Finnish Folk Song database^28^ and the Densmore database^29^. Thirty melodies were selected in total: seven melodies from North America (Native American), 13 from China, eight from Europe, and two from Africa. All were simple monophonic melodies. The number of selected melodies from each continent was uneven due to difficulties in finding African folk songs online consisting of only a simple, isolated melody, which was a requirement for maintaining consistency between musical stimuli, and for streamlining the extraction of the musical parameters.

To increase the experimental control, each melody was standardized using GarageBand v. 10.3.2 for Mac OS X. The tempo of each melody was set to 100 bpm and the key to a C (major or minor) so that the majority of notes for each melody fell within the same pitch register (C_3_ to C_4_). Some melodies were shortened in order to ensure equal length of melodies. In cases where the melody was incomplete due to cropping (i.e., stopping in the middle of a melodic phrase), the first note of the song was also added to the end, so that perceived incompleteness would not affect ratings. We acknowledge that this alteration in melodic structure may have further changed the cultural content of the music stimuli. In addition, to control for timbre, all melodies were rendered using a flute sound from the GarageBand library. Each melody lasted approximately 21 s.

#### Procedure

This study, together with Study 2, was approved by the Institutional Review Board (Internal Ethics Committee) at the Department of Psychology of the University of Oslo. The methods were carried out in accordance with relevant guidelines and regulations. After participants had accepted an invitation via Amazon Mechanical Turk, they were directed to a Qualtrics questionnaire where they received information about the study. Informed consent was obtained from all participants. They were then given instructions that they would listen to various songs from cultures that would be unknown to them. Participants were asked to use headphones to minimize distraction from the surrounding environment.

Next, participants listened to one melody at a time, presented in randomized order, and completed 7 VAS scales ranging from 0 to 100 for each melody. The ratings included musical warmth—“how warm/affectionate/kind does the music sound?” (*cold* to *warm*); musical threat—“how threatening does the music sound?” (*non-threatening* to *threatening*); musical energy—“how energetic is the music?” (*non-energetic* to *energetic*); musical liking—“how much do you like the music?” (*dislike* to *like*); musical happiness—“how happy does the music sound?” (*not happy at all* to *very happy*); musical advancement—“how advanced does the music sound?” (*primitive* to *advanced*); and musical familiarity—“how familiar does the music seem?” (*unfamiliar* to *familiar*). Participants also completed a multiple-choice question asking about the geographical region of the song—“where in the world do you think the melody is from?” (*Africa, Oceania, East Asia, Middle East, Eastern Europe, Western Europe, North America,* or *South America;* Answers to this question were used for stimulus selection for Study 2). Here and elsewhere, the presentation order of the scales was randomized for each participant.

Thereafter, participants were provided with a short text stating that the music excerpts they had listened to came from different, relatively unknown cultures. They were then given instructions that they would listen to all the excerpts one more time and rate the culture that the music stemmed from on six VAS scales after each melody. The melodies were again presented in a new randomized order. Listening to each song, participants rated the culture in terms of cultural warmth—“how warm/affectionate/kind does the culture seem?” (*cold* to *warm*); cultural competence—“how competent does the culture seem?” (*primitive* to *advanced*); cultural threat—“how threatening does the culture seem?” (*non-threatening* to *threatening*); cultural liking—“how much do you think you would like the culture?” (*dislike* to *like*); cultural happiness—“how happy does the culture seem?” (*not happy at all* to *very happy*); and cultural evolvedness (*ascent of man scale:* “Using the slider below, indicate how evolved you consider the culture to be”)*.* The standardized Ascent of Man Scale^15^ is a validated measure of blatant dehumanization that depicts five silhouettes illustrating five different stages of human evolution from apes to humans. Respondents can use a slider bar to indicate which of the five stages they consider a human culture or group to be at. We acknowledge that the “Ascent of Man” scale is contentious due to two conflicting motives. Whereas it highlights the severity of overt dehumanization as a detrimental intergroup consequence, enabling researcher to directly investigate its causes and potential remedies, there is also a shared apprehension among some participants and researchers about its use. One worry is that the scale may unintentionally foster dehumanization, but this has not been empirically supported so far^30^. Exact participant instructions and the full materials for Study 1 can be found in Appendix A.

#### Analyses

**Figure 1:**
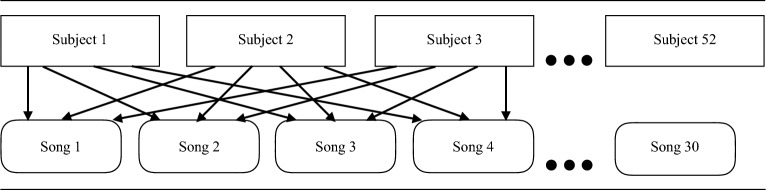
Crossed random effects design of Study 1.

The present study employed a crossed random effects design, which is illustrated in Fig. 1. Zero-order correlation analyses were first conducted to investigate the correlation between musical and cultural ratings of the song stimuli. These ratings were then standardized (*z*-scored) to increase the interpretability of effect sizes.

Two sets of analyses were conducted. First, the musical parameters of the melodies were extracted using the MIDI toolbox v. 1.1^31^ and the MIR-toolbox v. 1.7.2^32^ for MATLAB. Detailed parameter descriptions can be found in Table 1. Second, linear mixed model analyses were conducted to create 11 models: seven models testing the effects of musical parameters on musical ratings, and four models testing how musical parameters would influence cultural evaluations. Intercepts were set to random for participants and songs. Slopes were initially set to random for respective within-participant and within-song predictors (i.e., musical ratings); however, the models did not converge as they became too complex for the data. The final models therefore incorporated random intercepts, but not random slopes. The model equations can be found in Supplementary data (Appendix B). Analyses were performed using the *lme4* R package v. 1.1–23^33^ for modelling, and the *lmertest* R package v. 3.1–2^34^ to obtain p-values.

**Table 1:** Musical parameter descriptions.

Musical Parameters	Descriptions
Originality	This parameter refers to melodic complexity. The algorithm by Simonton^16^ is based on tone-transition probabilities. He analysed the first 6 notes of 15,618 melodies, and measured the frequency of combinations of notes occurring, thereby arriving at note transition probabilities. The less probable a combination is, the more original it is considered to be. The formula is provided as a function in the MIDI-toolbox^a^
GPV	This parameter refers to the general number of pitch classes (tones) used to construct the melody. If a melodic phrase consists of the notes c–c–f, this equals to a GPV-value of 2. This value is used as a measure of pitch variation. As the number of pitch classes increases, more variation is introduced to the melody
Interval Size	This parameter is a measure of distance between successive tones. It is a linear scaling of interval distributions, in which larger intervals have larger values than smaller ones. Each interval frequency (percentage of the melody) is multiplied by the number of semitones within that interval, and then summed together. For example, if the major second has a frequency value of 20 percent (i.e., making up 20% of the intervals in a melody), it is given the value of 0.4: 2 (semitones) × 0.2. Interval size distribution is provided as a function in the MIDI-toolbox^b^ Scaled function: *q* (*I*) =*|I| *× * p* (*I*), where *p* (*I*) refers to the probability of the interval occurring
Melodic Velocity	This parameter refers to the number of pitch onsets. The higher the number of pitch onsets, the shorter the interonset intervals. The parameter is a measure of ‘event density’ of the melody; the number of notes used in a melody within a given number of beats. The formula is inspired by Christensen and Nettl’s^35^ conceptualization of melodic tempo: melodic velocity = number of pitch onsets × 60/duration of melody in seconds. It should be noted that melodic velocity is measured within a set number of beats, and is therefore independent from tempo
Mode	This refers to estimation of mode, i.e., major vs. minor, returned as a numerical value between −1 and + 1: the closer it is to + 1, the more major the given excerpt is predicted to be, the closer the value is to −1, the more minor the excerpt is. The formula is provided as a function in the MIR-toolbox^b^

*GPV =* General pitch variation.

^a^Toiviainen and Eerola^31^.

^b^Lartillot and Toiviainen^32^.

### Results

The main analyses are presented in this section. Additional analyses (musical ratings on cultural evaluation) are presented in Appendix C. Unstandardized correlations, means and standard deviations for the musical and cultural ratings are displayed in Table 2.

**Table 2:** Unstandardized means, standard deviations and Pearson zero-order correlation coefficients for continuous variables.

	*M*	*SD*	1	2	3	4	5	6	7	8	9	10	11
1. Musical Warmth	53.07	22.79	–										
2. Musical Threat	23.29	22.68	−0.52**	–									
3. Musical Liking	54.91	22.32	−0.56**	−0.35**	–								
4. Musical Happiness	47.22	47.22	−0.71**	−0.46**	0.50**	–							
5. Musical Energy	47.94	24.67	0.58**	−0.33	0.50	0.72**	–						
6. Musical Advancement	50.49	22.14	0.37*	−0.17	0.46	0.42*	0.49*	–					
7. Musical Familiarity	40.19	26.22	0.30*	−0.18*	0.41*	0.26*	0.22*	0.25	–				
8. Cultural Warmth	56.23	23.08	0.46**	−0.37**	0.36**	0.48**	0.44*	0.30	0.15*	–			
9. Cultural Threat	27.65	24.56	−0.37**	0.36**	−0.27**	−0.32**	−0.28**	−0.18	−0.04*	−0.52**	–		
10. Cultural Competence	63.18	23.06	0.33*	−0.28**	0.36**	0.36*	0.37**	0.31*	0.25	0.48**	−0.28**	–	
11. Cultural Evolvedness	80.65	21.76	0.36**	−0.30**	0.38**	0.38**	0.37**	0.34*	0.04	0.52**	−0.36**	0.69**	–

N = 30. Responses ranged from 0 to 100.

*M =* Mean, *SD =* Standard deviation.

*p < .05. **p < .01. ***p < 0.001.

#### Effect of objective musical parameters on musical ratings

The standardized associations of objective musical parameters with subjective musical ratings can be found in Table 3. Originality was positively associated with perceived musical liking (*d* = 0.2) and musical advancement (*d* = 0.1). General pitch variation was positively associated with perceived musical energy (*d* = 0.1) and negatively with perceived musical happiness (*d* = −0.1). Interval size was positively associated with perceived musical happiness (*d* = 0.2).

**Table 3:** Standardized regression coefficients for musical ratings on musical parameters.

Variables	Warmth Intercept = 0.000 *SE* = 0.071 *df* = 56.36 t = 0.000 *p* = 0.999	Threat Intercept = 0.000 *SE* = 0.090 *df* = 68.74 t = 0.000 *p* = 0.999	Liking Intercept = 0.000 *SE* = 0.081 *df* = 64.86 t = 0.000 *p* = 1.00	Happiness Intercept = 0.000 *SE* = 0.072 *df* = 50.64 t = 0.000 *p* = 0.999	Energy Intercept = 0.000 *SE* = 0.064 *df* = 51.33 t = 0.000 *p* = 0.999	Advanced Intercept = 0.000 *SE* = 0.082 *df* = 62.92 t = 0.000 *p* = 1.00	Familiarity Intercept = 0.000 *SE* = 0.104 *df* = 62.47 t = 0.000 *p* = 0.999
Originality	0.045 (0.069)	0.020 (0.074)	**0.142 (0.057)**	0.014 (0.076)	0.058 (0.071)	**0.011 (0.053)**	0.086 (0.057)
	*p* = 0.512	*p* = 0.793	***p* = 0.020**	*p* = 0.857	*p* = 0.410	***p* = 0.004**	*p* = 0.148
			***d* = 0.2**			***d* = 0.1**	
GPV	−0.029 (0.058)	0.003 (0.096)	0.024 (0.074)	**−0.056 (0.063)**	**0.183 (0.092)**	0.045 (0.068)	−0.062 (0.075)
	*p* = 0.619	*p* = 0.977	*p* = 0.744	***p* = 0.023**	***p* = 0.047**	*p* = 0.518	*p* = 0.409
				***d* = −0.1**	***d* = 0.1**		
Interval Size	0.060 (0.071)	−0.114 (0.089)	0.070 (0.069)	**0.067 (0.078)**	0.141 (0.086)	0.055 (0.064)	−0.000 (0.069)
	*p* = 0.404	*p* = 0.214	*p* = 0.317	***p* = 0.002**	*p* = 0.112	*p* = 0.397	*p* = 0.999
				***d* = 0.2**			
Melodic Velocity	**0.401 (0.057)**	**−0.184 (0.057)**	**0.283 (0.044)**	**0.525 (0.632)**	**0.604 (0.055)**	**0.298 (0.040)**	0.072 (0.044)
	***p* < 0.001**	***p* = 0.003**	***p* < 0.001**	***p* < 0.001**	***p* < 0.001**	***p* < 0.001**	*p* = 0.119
	***d* = 0.5**	***d* = −0.2**	***d* = 0.4**	***d* = 0.6**	***d* = 0.8**	***d* = 0.4**	
Mode	**0.160 (0.056)**	**−0.152 (0.055)**	0.002 (0.043)	**0.113 (0.061)**	0.054 (0.053)	0.037 (0.040)	−0.051 (0.043)
	***p* = 0.009**	***p* = 0.011**	*p* = 0.99	***p* < 0.001**	*p* = 0.318	*p* = 0.355	*p* = 0.253
	***d* = 0.2**	***d* = −0.2**		***d* = 0.2**			

Standard deviations in parentheses. Significance below 0.05 level in bold. Effect size (Cohen’s d) in bold for significance below 0.05.

*GPV =* General pitch variation, *Mode =* Major mode.

Furthermore, melodic velocity was positively associated with perceived musical warmth (*d* = 0.5), musical liking (*d* = 0.4), musical happiness (*d* = 0.6), musical energy (*d* = 0.8), and musical advancement (*d* = 0.4), and negatively with perceived musical threat (*d* = −0.2). Finally, mode was positively associated with perceived musical warmth (*d* = 0.2) and musical happiness (*d* = 0.2) and negatively with perceived musical threat (*d* = −0.2).

#### Effect of objective musical parameters on culture evaluations

The standardized association between objective musical parameters and subjective cultural evaluations can be found in Table 4. Originality was positively associated with cultural competence (*d* = 0.2). Interval size was positively associated with perceived cultural warmth (*d* = 0.2). Melodic velocity was positively associated with perceived cultural warmth (*d* = 0.5), cultural competence (*d* = 0.3) and cultural evolvedness (*d* = 0.3) and negatively with perceived cultural threat (*d* = −0.2). Finally, mode was positively associated with perceived cultural warmth (*d* = 0.2) and negatively with perceived cultural threat (*d* = −0.2).

**Table 4:** Standardized regression coefficients for cultural ratings on musical parameters.

Variables	Warmth Intercept = 0.000 *SE* = 0.073 *df* = 55.47 t = 0.000 *p* = 0.999	Threat Intercept = 0.000 *SE* = 0.090 *df* = 68.74 t = 0.000 *p* = 0.999	Competence Intercept = 0.000 *SE* = 0.090 *df* = 66.90 t = 0.000 *p* = 0.999	Evolvedness Intercept = 0.000 *SE* = 0.109 *df* = 58.26 t = 0.000 *p* = 0.999
Originality	0.047 (0.073)	0.019 (0.074)	**0.107 (0.063)**	0.065 (0.047)
	*p* = 0.524	*p* = 0.657	***p* = 0.021**	*p* = 0.127
			***d* = 0.2**	
GPV	−0.077 (0.060)	0.002 (0.095)	−0.001 (0.082)	−0.024 (0.061)
	*p* = 0.217	*p* = 0.977	*p* = 0.989	*p* = 0.787
Interval Size	**0.114 (0.074)**	−0.114 (0.089)	0.058 (0.076)	0.037 (0.057)
	***p* = 0.027**	*p* = 0.214	*p* = 0.340	*p* = 0.189
	***d* = 0.2**			
Melodic Velocity	**0.390 (0.060)**	**−0.120 (0.057)**	**0.237 (0.048)**	**0.194 (0.037)**
	***p* < 0.001**	***p* = 0.003**	***p* < 0.001**	***p* < 0.001**
	***d* = 0.5**	***d* = −0.2**	***d* = 0.3**	***d* = 0.3**
Mode	**0.137 (0.059)**	**−0.149 (0.055)**	0.030 (0.047)	0.036 (0.035)
	***p* = 0.028**	***p* = 0.011**	*p* = 0.143	*p* = 0.340
	***d* = 0.2**	***d* = −0.2**		

Standard deviations in parentheses. Significance below 0.05 level in bold. Effect size (Cohen’s d) in bold for significance below 0.05.

*GPV =* General pitch variation, *Mode =* Major mode.

### Discussion

The results demonstrated how musical parameters and perceptions are linked to cultural evaluations. Melodic velocity—a characteristic seldom focused on in previous work—influenced musical and cultural evaluations, with higher melodic velocity associated with positive evaluations in both the musical and cultural domains. Melodic velocity, although related to the number of events within a given time window, is conceptually different from tempo, as tempo was held constant. Increased melodic velocity reflects increased number of pitch onsets, which may be associated with increased complexity and/or increased arousal (as found when increasing tempo;^36,37^. Increased arousal may provide people with a richer and more pleasing experience associated with positivity and happiness^38^, feelings that in turn seem to extend to the cultural source of that music.

Next, major mode was also associated with positive musical and cultural evaluations. However, this parameter exerted generally weaker effects than musical velocity. The current results complement previous findings on the association of the major mode with positivity and happiness^19–21^. Additionally, it also supports the finding that although the minor mode is associated with increased sadness, it is not necessarily less liked^24^. The current study extends previous findings and demonstrates that the major mode influences the perception of warmth in a culture and is not related to aspects of competence. The reason for this finding may be that the social feeling of warmth is associated with the positive feeling of happiness that the major mode reflects. On the other hand, happy and sad feelings, induced by major and minor mode music, respectively, may be perceived as equally complex, leaving the perceived competence of their cultural source unaffected.

In the current study, interval size was weakly, but significantly, associated with increased musical happiness and increased cultural warmth. This finding may indicate that the emotional connotations of happiness in larger intervals^17^ may also be associated with rating the associated culture higher in warmth, at least within this context and group under investigation. Higher general pitch variation was weakly associated with decreased happiness in music and increased energy of music. Moreover, higher originality was weakly associated with higher ratings of liking and advancement for the music, which is consistent with previous research on this parameter in relation to composer popularity^16^. The current study also found a relationship between higher musical originality and higher perceived cultural competence. Although weak in size, it may indicate that the unusualness of a melody is marginally related the perception of higher competence in a culture.

Using such controlled stimuli as in the present study increases internal validity but comes at the expense of excluding other culturally informative content from the melodies. As such, it could be argued that the participants heard and rated homogenized and arguably ‘Western’ presentations of music that are void of context and performer expression. However, by stating that the songs came from different, relatively unknown cultures, we have aimed to investigate whether a specific set of musical parameters can be associated with both musical and cultural evaluations in the absence of additional cultural information. As any methodological approach, it has strengths and disadvantages.

While providing initial insights, the current study only cross-sectionally investigated the musical parameters already present in the melodies without experimentally altering them. While this approach strengthens external validity, it does not provide information about the causality of effects. The next experimental study aims to address this limitation.

## Study 2

The second study set out to investigate the causal effect of musical parameters on cultural evaluations by retaining full control of parameters via manipulation. Tempo and dissonance were chosen for parameter manipulation as they allowed for alteration without changing the melodic structure in terms of pitch variation, pitch number or pitch direction. Additionally, these parameters have been extensively studied in relation to musical preference and emotional connotations. A number of studies have postulated that, in terms of emotional connotations, a fast tempo in music reflects happiness, whereas a slow tempo reflects sadness^6,21,39^. However, other studies have indicated that changes in tempo may be associated with changes in arousal rather than emotional valence, which in turn can also affect performance on various cognitive^36–38^ and behavioural tasks^40–42^. Increased tempo has also been associated with greater preference in the context of classical music^43^.

Moreover, there is general agreement that music intervals with simple frequency ratios, termed consonant, are considered preferable and associated with pleasure, whereas intervals with complex frequency ratios, termed dissonant, are considered non-preferable and associated with unpleasantness and fear among Western listeners^44,45^. The preference for consonance seems to be even stronger among musically educated and experienced Western listeners and correlates with years of musical training^46,47^.

By selecting a subset of folk songs from Study 1 and altering their tempo and consonance/dissonance, we tested the direct influence of these musical parameters on cultural evaluations. We expected that fast tempo would yield positive cultural evaluations based on previous work. Additionally, we expected that consonant melodies would predict positive cultural evaluations. Two broader categories of information processing theories are put forward in order to explain how the propensity for Western listeners to prefer consonant melodies could extend to preference for the cultural source of such music. First, the fluency concept describes the ease of processing stimuli^48^. Studies have found that fluent processing positively predicts social evaluations, which has led to the theorizing that ease of processing may also influence prejudice^49–51^. Second, the principle of similarity for social attraction may be explained by social identity^52^ and self-categorization theory^53^. This principle indicates that individuals who are perceived as more similar to oneself are more likely to be perceived as ingroup members and, in turn, more likely to be positively evaluated, unless similarity poses a threat to group distinctiveness^1^. Applied to the musical domain, research shows that shared musical preferences lead to more social attraction due to value similarity^54,55^. A recent study also found that the alteration from consonance to dissonance in the dynamic interplay between two performers decreased listeners’ ratings of positive social intentions related to affiliation^9^. Such an effect may also extend to the evaluation of cultures.

Moreover, considering the relationship between increased tempo and arousal^37^ and the relationship between dissonance and unpleasantness^56^, it was expected that an interaction between increased consonance and increased tempo would predict higher cultural warmth, whereas increased dissonance and higher tempo would predict higher cultural threat. Finally, it was expected that the positive effects of consonance and the negative effects of dissonance would be more pronounced for people who had received prior musical training, following work by Dellacherie et al.^46^ and McDermott et al.^47^.

### Methods

#### Participants

Similar to Study 1, participants were recruited through Amazon Mechanical Turk. The power rationale was the same as in Study 1; however, since we used manipulated song versions in four alterations, samples 200 participants in total, with approximately 50 participants for each alteration. An initial total of 270 participants were recruited. Of these participants, 212 (78 female, 134 male, age range = 20–74, *M*_*age*_ = 38.51, *SD*_*age*_ = 11.12) fulfilled the inclusion criteria. Similar to Study 1, participants were selected based on their ethnicity (White/Caucasian American) to minimize the variability in the degree of cultural familiarity and affiliation with the folk songs selected.

#### Musical stimulus selection

Melodies were selected on the basis of the geographic location ratings obtained in Study 1. The melodies with the highest standard deviation in terms of geographic location were selected for the present study. The aim of this approach was to select those melodies that were the most difficult to locate, thus resulting in less systematic pre-existing bias towards their cultural origins. The standard deviations of the geographical region in Study 1 ranged from 1.40 to 2.88. Eight melodies were selected with a cut-off standard deviation of 2.30. Two of these songs were discarded. First, the song with the highest standard deviation (2.88) only contained two pitches, making it impossible to create dissonant intervals without moving a pitch more than one semitone. Second, the melody with the third highest standard deviation (2.47) contained a melodic structure that made it unfeasible to create dissonant intervals without changing more than one pitch. Therefore, six melodies were retained as stimuli for this study.

**Figure 2:**
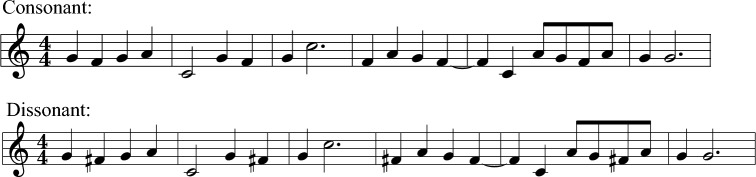
Difference between a consonant and a dissonant version of a melody. Note: Excerpt from the first 6 bars of song nr. 14. The original (top) originated in the Han dynasty in China (206 BC-220 AD).

We created four versions of each melody by manipulating tempo and dissonance using GarageBand v. 10.3.2 and Logic Pro X v. 10.4.8 for Mac OS X, resulting in consonant-slow, consonant-fast, dissonant-slow, and dissonant-fast versions of each melody. To create slow and fast versions of each melody, the tempo was either increased 20 bpm or reduced 20 bpm, resulting in tempos of 80 bpm and 120 bpm (respectively) while keeping pitch constant. To create dissonant versions of each melody (in addition to the original consonant version), we used a system where one pitch class in the melody was transposed either a semitone up or down, thereby creating a certain number of dissonant intervals in the melody. The dissonant intervals created were either a major second changed to a semitone or a perfect 4^th^ or perfect 5^th^ changed to a Tritone (with reference to the tonic; see Table 5 for details). An example notation excerpt of the difference between a consonant and dissonant version of the same melody is illustrated in Fig. 2.

**Table 5:** Dissonance manipulations.

	Location	Bars	Pitch class moved	Interval with the tonic (C)	% of dissonant intervals	% of semiquavers moved	*SD* of location
Song 2	America	10	F to F♯	Tritone	17.9	20.6	2.31 (8)
Song 3	America	9	D to D♭	Semitone	68.4	32.5	2.38 (5)
Song 6	America	8	D to D♭	Semitone	35.7	31.3	2.36 (6)
Song 14	China	9	F to F♯	Tritone	25.9	20.8	2.39 (4)
Song 15	China	9	G to G♭	Tritone	21.1	31.6	2.31 (7)
Song 29	Africa	8	F to F♯	Tritone	44.4	21.4	2.52 (2)

Percentage of semiquavers refers to the proportion of sixteenth note units that were moved to create dissonance. 1 moved quarter note (crotchet) equals 4 moved semiquavers. Ranking from the highest to lowest standard deviation of geographical location estimates in parentheses.

#### Procedure

This study, together with Study 1, was approved by the Institutional Review Board (Internal Ethics Committee) at the Department of Psychology of the University of Oslo. The methods were carried out in accordance with relevant guidelines and regulations. All participants were informed about the study and agreed to participate. The procedure was similar to Study 1. Each participant listened to each of the six melodies once and evaluated the cultural origin of the melody on the six rating scales from the previous study (warmth, threat, happiness, liking, competence and evolvedness). Importantly, for each song, the participants were randomly assigned to listen to one of four possible variations: consonant-fast, consonant-slow, dissonant-fast or dissonant-slow. This was randomized for each song. Participant instructions for Study 2 can be found in Appendix A.

Finally, participants also completed a demographic questionnaire recording their gender, age and musical training. The study took approximately 7 min to complete, and participants were awarded $1 for participation.

#### Analyses

**Figure 3:**
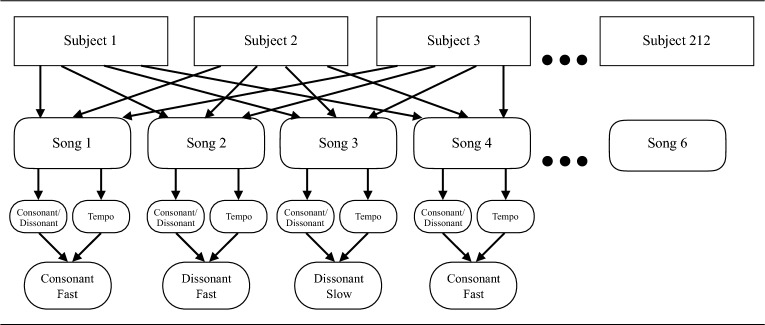
Crossed random effects design of Study 2. Note: Random presentation of dissonance and tempo between songs (bottom level in figure).

The models specified a crossed random effects design that represented the data structure and is illustrated in Fig. 3. In factorial terms, a 2 (tempo: slow versus fast) X 2 (consonance versus dissonance) factorial design was utilized.

All variables were *z*-scored to ease interpretation. Similar to Study 1, linear mixed model analyses were conducted to create 10 models. The first four models aimed to investigate the effect of dissonance and tempo on cultural ratings. These models also tested whether there was an interaction effect between the two parameters. In addition, due to the high number of musically trained participants in the sample (33%), we explored group differences between people with musical training and people without musical training. We defined musical training as having actively played an instrument for 2 years or more. Specifically, six additional models aimed to investigate whether musical training could affect cultural evaluations when controlling for other individual characteristics. Cultural liking and perceived cultural happiness were added as dependent variables to examine a general trend in cultural ratings by musically trained vs. untrained participants. Moreover, to test our prediction that musically trained individuals experienced greater preference for consonance and greater aversion to dissonance, the models examined whether musical training interacted with dissonance in predicting cultural evaluations in a second model step.

To rule out potential confounding variables in the main effects or interaction, we added gender and age to the models in a third model step. Participants and songs were entered as random intercepts. They were also entered as random slopes for within-participant and within-song variables. All analyses were performed using the same packages as in Study 1. Each factor variable (tempo and dissonance vs. consonance) was contrast-coded so that the results could be interpreted with reference to the grand mean. Boxplots were created for visualization using the *ggplot2* R package v. 3.3.2^57^. The model equations can be found in Appendix B.

### Results

The main analyses are presented in this section. Additional analyses (degree of dissonance on cultural evaluations) are presented in Appendix C.

#### Effects of dissonance and tempo on cultural evaluations

**Figure 4:**
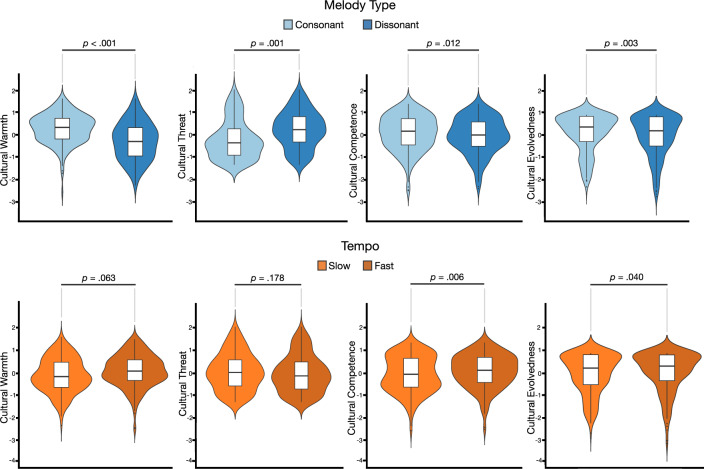
Response distributions and boxplots of mean cultural ratings by subject. Note: Vertical lines (whiskers) in boxplots indicate entire distribution of responses within 1.5 times the interquartile range above the 75th percentile and below the 25th percentile. The upper and lower horizontal lines represent the interquartile range from the 75th percentile to the 25th percentile. The horizontal line in the middle represents the median.

The standardized regression estimates of subjective cultural evaluations on dissonance and tempo can be found in Table 6. Participants’ cultural ratings for consonant and dissonant melodies and slow and fast tempo are illustrated in Fig. 4. There was a significant positive effect of dissonance on perceived cultural threat (*d* = 0.7) and a significant negative effects of dissonance on perceived cultural warmth (*d* = −0.8), cultural competence (*d* = −0.2) and cultural evolvedness (*d* = −0.2). Moreover, there were significant positive effects of tempo on perceived cultural competence (*d* = 0.2) and cultural evolvedness (*d* = 0.1). In a second model step, an interaction term was added to the model. There were no interaction effects of dissonance and tempo on any of the cultural ratings.

**Table 6:** Standardized regression coefficients for cultural ratings on musical parameters.

	Variables	Warmth Intercept = −0.008 *SE* = 0.089 df = 6.99 t-value = −0.090 *p* = 0.931	Threat Intercept = 0.000 *SE* = 0.079 df = 14.68 t-value: −0.001 *p* = 0*.*998	Competence Intercept = 0.000 *SE* = 0.100 df = 7.62 t-value: −0.007 *p* = 0.995	Evolved Intercept = −0.003 *SE* = 0.079 df = 16.03 t-value = −0.038 *p* = 0*.*969
Step 1	Dissonance	**−0.557 (0.084)**	**0.453 (0.099)**	**−0.112 (0.046)**	**−0.127 (0.042)**
		***p* < 0.001**	***p* = 0.005**	***p* = 0.012**	***p* = 0.003**
		***d* = −0.8**	***d* = 0.7**	***d* = −0.2**	***d* = −0.2**
	Fast Tempo	0.190 (0.049)	−0.080 (0.052)	**0.137 (0.049)**	**0.080 (0.038)**
		*p* = 0.063	*p* = 0.178	***p* = 0.006**	***p* = 0.040**
				***d* = 0.2**	***d* = 0.1**
Step 2	Dissonance: Fast Tempo	0.027 (0.091)	0.032 (0.078)	0.066 (0.090)	0.052 (0.075)
		*p* = 0.768	*p* = 0.683	*p* = 0.463	*p* = 0.492

Categorical predictors are contrast coded. Standard deviations in parentheses. Significance above 0.5 in bold. Effect size (Cohen’s d) in bold for significance below 0.05.

#### Effect of individual differences on culture evaluations

**Figure 5:**
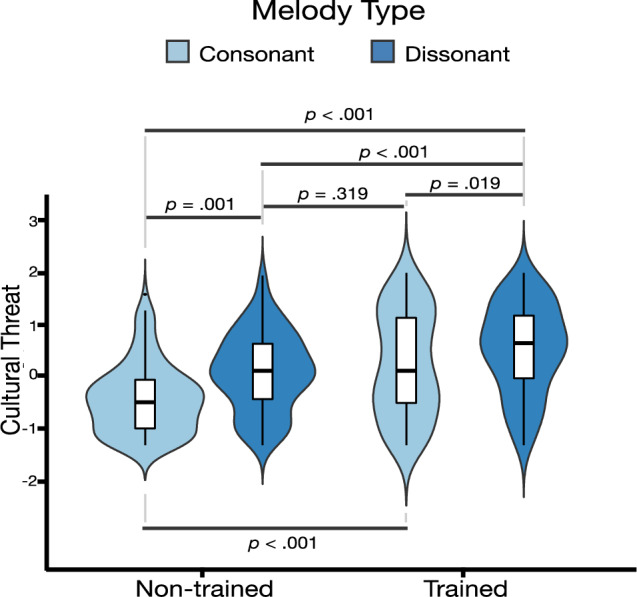
Response distributions and boxplots of mean cultural threat ratings for consonant and dissonant melodies among musically trained and non-trained participants. Note: Vertical lines (whiskers) in boxplots indicate entire distribution of responses within 1.5 times the interquartile range above the 75th percentile and below the 25th percentile. The upper and lower horizontal lines represent the interquartile range from the 75th percentile to the 25th percentile. The horizontal line in the middle represents the median.

The regression estimates for subjective cultural evaluations on musical training and demographic variables can be found in Table 7. There was a significant positive effect of musical training on perceived cultural threat (*d* = 0.9) and perceived cultural competence (*d* = 0.4). As we suspected that the higher perception of perceived threat and perceived competence by people with musical training was due to these subjects generally experiencing more intense affect through music, we tested whether people with musical training also rated the cultures as happier and more likable in two additional models. Indeed, there was a significant positive effect of musical training on perceived cultural liking (*d* = 0.4) and perceived cultural happiness (*d* = 0.4). In a second model step, we added dissonance as a predictor and included an interaction term in the models. We found a significant negative interaction effect between dissonance and musical training on perceived cultural threat (*d* = −0.3). Pairwise comparisons revealed that the effects of consonance and dissonance on cultural threat ratings were smaller for people with musical training (β = −0.32, *p* = 0.019) than for people without musical training (β = −0.52, *p* = 0.001). The interaction is illustrated in Fig. 5. To rule out any confounding variables in the main effects of musical training or interaction between musical training and dissonance, we added gender and age to these models in a third model step. There was a significant main effect of age on perceived cultural threat (*d* = −0.2), but a fourth model step revealed no interaction between musical training and age, ruling out these characteristics as confounders.

**Table 7:** Standardized regression coefficients for cultural ratings on musical training and demographics.

	Variables	Warmth Intercept = −0.053 *SE* = 0.077 df = 7.99 t-value = 0.687 *p* = 0.511	Threat Intercept = 0.081 *SE* = 0.079 df = 12.97 t-value = 1.01 *p* = 0.328	Competence Intercept = 0.073 *SE* = 0.096 df = 8.67 t-value = 0.767 *p* = 0.463	Evolved Intercept = 0.060 *SE* = 0.079 df = 11.86 t-value = 0.754 *p* = 0*.*465	Liking Intercept = 0.058 *SE* = 0.091 df = 4.42 t-value = 0.637 *p* = 0*.*556	Happiness Intercept = 0.039 *SE* = 0.079 df = 16.03 t-value = −0.038 *p* = 0*.*708
Step 1	Musical Training	0.202 (0.107)	**0.563 (0.103)**	**−0.117 (0.108)**	*p* = 0.189	**0.289 (0.107)**	**0.222 (0.122)**
		*p* = 0.058	***p* < 0.001**	***p* = 0.005**	*p* = 0.189	***p* = 0.009**	***p* = 0.004**
			***d* = 0.9**	***d* = 0.4**		***d* = 0.4**	***d* = 0.4**
Step 2	Dissonance	**−0.535 (0.087)**	**0.421 (0.094)**	**0.314 (0.108)**	**−0.137 (0.121)**	**−0.423 (0.072)**	**−0.508 (0.072)**
		***p* < 0.001**	***p* = 0.006**	***p* = 0.013**	***p* = 0.024**	***p* < 0.001**	***p* < 0.001**
	Musical Training: Dissonance	0.145 (0.113)	**−0.195 (0.094)**	−0.035 (0.095)	−0.071 (0.088)	0.005 (0.102)	−0.005 (0.110)
		*p* = 0.205	***p* = 0.040**	*p* = 0.710	*p* = 0.420	*p* = 0.960	*p* = 0.964
			***d* = −0.3**				
Step 3	Gender	–	0.112 (0.102)	−0.154 (0.103)	–	−0.114 (0.102)	−0.071 (0.101)
			*p* = 0.270	*p* = 0.149		*p* = 0.266	*p* = 0.709
	Age	–	**−0.157 (0.049)**	0.021 (0.048)	–	−0.054 (0.046)	−0.046 (0.047)
			***p* = 0.003**	*p* = 0.836		*p* = 0.244	*p* = 0.338
			***d* = −0.2**				
Step 4	Musical Training: Age	–	−0.012 (0.104) *p* = 0.905	–	–	–	–

Categorical predictors are contrast coded. Standard deviations in parentheses. Significance above 0.5 in bold. Effect size (Cohen’s d) in bold for significance below 0.05.

### Discussion

Fast tempo predicted evaluations of cultural competence and cultural evolvedness, indicating that participants perceived the cultural source of music at tempo 120 bpm as being more competent and evolved than at 80 bpm. The fact that tempo influenced perceived cultural competence and not cultural warmth or cultural threat may have to do with people’s general association of competence with the speed with which a task is performed. As one develops competence in a skill, the speed at which the skill is executed will usually increase^58,59^. Moreover, faster tempo is by definition an increase in musical speed, which is also associated with increased bodily speed on behavioural tasks, such as walking^40^, reading^41^ and driving^42^. The tempo findings also align with the melodic velocity results in Study 1, suggesting that increased event density may also be experienced as more complex and/or more arousing. This finding is not surprising considering how the notion of virtuosity is conceptualized in Western societies, which recognises famous, virtuosic European concert pianists as exhibiting great skill, speed and accuracy^60^, which in turn are considered a product of hard work by Western music professionals and students^61^. Thus, musical speed may have other perceptual downstream consequences in different cultural contexts.

In support of our predictions, dissonance significantly predicted all cultural ratings. Specifically, participants found the cultural source of consonant melodies warmer and the cultural source of dissonant melodies more threatening. These strong effects further indicate that dissonance in music can be an important factor in the evaluation of warmth and threat by a culture. Our findings also support those of Aucouturier and Canonne^9^, who demonstrated that increased dissonance led to lower ratings of positive social affiliation.

From a fluency perspective, consonance may be perceived by Western participants as warmer and less threatening due to consonance being easier to cognitively process as a function of increased familiarity. This interpretation is consistent with previous studies indicating that the ease of communication and increased previous exposure both promote positive outgroup evaluations^49–51^. Furthermore, the finding that consonant music predicted more positive cultural ratings may indicate that Western listeners prefer intervals that are more commonly used in their own culture^1^.

People with musical training gave higher ratings of threat, competence, liking and happiness for the cultures compared to individuals without musical training. Consistent with this, musically experienced individuals have been shown to experience more arousal in response to sad and scary music^62,63^. Musical expertise is also related to greater accuracy in recognizing emotions in music^64^. The finding that musically trained individuals found cultural sources of music more threatening and more likable, competent and happy may therefore reflect the fact that musically trained individuals may be more sensitive to expressive and communicative nuances in music. It is also possible that musically trained participants are more aware of the stylistic norms of Western music, and potentially less affected by these norms in the context of evaluating the music of unknown cultures. It is important to note, however, that the current study did not assess the degree or type of musical training received.

Furthermore, musical training negatively interacted with dissonance in predicting threat. Specifically, people with musical training were less affected by the consonance-dissonance distinction in evaluating threat. This finding was somewhat surprising and countered our predictions^46,47^. It may be that although people with musical training experience greater unpleasantness and aversion to dissonance, they also have more experience with dissonant intervals and therefore have a more objective view of consonance and dissonance when applying them to a cultural context. Musically trained individuals may also be more aware of varying stylistic conventions (such as differential preference for consonance) between different musical cultures, for example. Consonance, which may be interpreted as an indication of similarity to Western culture, may be a weaker elicitor of positive outgroup attitudes if moderators such as high ingroup identification or high dimension importance are present^1^. However, future research is needed to directly test this interpretation.

Finally, contrary to predictions, there were no interaction effects of tempo and dissonance on either threat or warmth. However, due to the short duration of the melodies, limited variation in dissonance and only two tempo variations, we cannot affirm that such a relationship does not exist. It could be that in evaluating cultural sources of music, the interval structure is a more prominent indicator of warmth and threat regardless of tempo alterations. Thus, even though a faster tempo may lead to higher arousal, this elevation in arousal may not necessarily change the pattern of warmth/threat associations related to dissonance.

## General discussion

The present findings extend previous research on the effects of musical parameters on personal preference and emotional connotations to the domain of broader social evaluations. The indirect contact hypothesis states that prejudice can be reduced by indirect intergroup contact, such as through television or books^65,66^. Similarly, music has been shown to have the potential to alter cultural biases^2^. Thus, music as a medium may communicate content that can change people’s social evaluations. At the same time, it is important to note that our results are not universal and need to be interpreted within their specific cultural context. The present research revealed that formal musical parameters, such as velocity, mode, tempo and interval structures, are associated with Western listeners’ preferences and ratings of other cultures in the absence of other musical or cultural context. Furthermore, we demonstrated that the alteration of such parameters can affect Western listeners’ ratings on several dimensions of intergroup evaluations. Whether the alteration of such parameters can actually change people’s real-life evaluations of other cultures in both positive and negative directions is an important aspect to consider in future studies when evaluating music as a potential candidate for increasing rapport between cultures. Additionally, as in any research aiming to explain intergroup perceptions and biases, it is important to note that the observations in our studies are a product of participants’ pre-existing biases interacting with the type of stimuli they are exposed to. As such, the biases observed here are inherently cultural, as they took place in a distinct cultural setting and a distinct cultural group. Moreover, many previous studies have studied music in conjunction with lyrics, making it difficult to disentangle their individual effects^67^. The current research focused on musical parameters specifically in the absence of lyrical content and may therefore aid future studies in understanding how specific musical phenomena may influence social phenomena.

Even though a highly controlled study design allowed us to minimize the potential effects of extraneous variables, it also posed some limitations to the present research. First, since all our participants identified as white/Caucasian American, the present findings only reflect the responses of a specific group of Western enculturated listeners. Since musical conventions and their connotations vary from culture to culture, it is possible that listeners from a different cultural background might interpret certain musical parameters differently. Future, cross-cultural research would be well-positioned to test this possibility. Such studies could for instance investigate whether dissonant intervals create the same judgements of threat across different ethnolinguistic groups with varying exposure to Western media. This research could have important implications for the nature-nurture debate on the preference for consonance, following work by McDermot et al.^68^, broadening the scope from preference to social functionality of the consonance-dissonance distinction.

Second, although we attempted to include musical examples from different parts of the world, some world regions were better represented than others. However, the main aim of the stimulus selection was to find sufficiently varied musical material that would be unfamiliar to the participants, somewhat alleviating this concern. Third, while we assessed a relatively high number of musical parameters, our selection was not exhaustive. For instance, it could be interesting to investigate the role of other parameters such as timbre, pitch, interplay, intonation or harmony; however, these were considered beyond the scope of the present research, which only focused on simple monophonic melodies. Additionally, the type of music-listening setting utilized in the current studies, lacking any context to place the presented music in, does not reflect actual music-listening experiences in the real world. Thus, the drawback of the present work is that it may have high internal control but lack ecological validity in terms of musical engagement and consumption.

Fourth, although we aimed to use highly controlled song stimuli, one way to ensure even higher experimental control could be to eliminate the flute timbre, as this timbre is likely to interact with pre-existing notions and associations related to both music and cultures. The direct effect of the flute timbre, and even potential interaction effects between the flute timbre and melody, could thus not be controlled for in the present research. Future research should test for the influence of different musical instruments on cultural evaluation. Finally, one must keep in mind that real-life perceptions of cultures may be influenced by repeated exposure to full-length complex songs and the identification with their performers. Thus, the small effects we observed, although statistically significant and consistent, may be partly due to the short, simplified MIDI excerpts that only used the flute timbre. Future research may test this possibility with more naturally-occurring stimuli. Such research should also investigate the degree to which musical parameters may interact with other types of information, such as individual characteristics and the context of music listening.

### Conclusion

In two studies, we show how objective parameters in music are associated with (Study 1) or predict (Study 2) people’s subjective ratings of the music and its cultural source. In Study 1, increased melodic velocity, a measure of melodic event density, had a particularly consistent positive effect on musical and cultural evaluations. In Study 2, the cultural source of music was perceived as warmer and less threatening when the music was consonant rather than dissonant. Music may have the potential to reduce prejudice.

### Supplementary Information

Supplementary Information.

## Data Availability

All stimuli and datasets analysed in this study can be found in an online repository: https://www.osf.io/a3npz/.
