# Supplementary material for: The influence of musical parameters and subjective musical ratings on perceptions of culture
Source: Sci Rep. 2023 Nov 24;13:20682. doi: 10.1038/s41598-023-45805-w (PMC10673861; doi:10.1038/s41598-023-45805-w)
Supplement: Supplementary file 1 — Supplementary Information. [file 41598_2023_45805_MOESM1_ESM.pdf]

Supplementary material

Appendix A. Participant instructions for Study 1 and 2

Instructions for Study 1 part 1 were as follows:

“Thank you for choosing to participate in this study.

This study has 2 parts in which you will be presented with 60 music excerpts. In the first part you will be presented 30 music excerpts. After listening to each music excerpt your task is to rate each of these melodies, using multiple choice and the slider bars on eight domains.

Please remember to use headphones in order to minimize input from other sounds nearby.

The domains are:

Warmth - how warm/affectionate/kind does the music sound?

Advancement - how advanced does the music sound?

Threat - how threatening does the music sound?

Energy - how energetic is the music?

Liking - how much do you like the music?

Happiness - how happy does the music sound?

Familiarity - how familiar does the music seem?

Geographical region - where in the world do you think the melody is from?

Please click the play button to listen to the song. When the song is finished, answer the following questions.”

Instructions for Study 1 part 2 were as follows:
“Thank you for completing the first part of this study.
The music excerpts you listened to actually came from different, relatively unknown cultures. In the next part of the study, we would like you to listen to the excerpts once more, this time rating the culture they come from, based on the music you hear. Try to base your ratings on what the music itself conveys, and not where you think the music comes from.

The domains are:

Warmth - how warm/affectionate/kind does the culture seem?

Competence - how competent does the culture seem?

Threat - how threatening does the culture seem?

Liking - how much do you think you would like the culture?

Happiness - how happy does the culture seem?

Evolved - how evolved do you consider the culture to be?

Please click the play button to listen to the song. When the song is finished, answer the following questions."

Instructions for Study 2 were as follows:
"Thank you for choosing to participate in this study.

In this study you will be presented 6 music excerpts from various relatively unknown cultures. After listening to each music excerpt your task is to rate each of these melodies based on your impression of the culture the music stems from, using the slider bars on six domains. Try to base your ratings on what the music itself conveys, and not on any existing cultures that the music may remind you of.

Please remember to use headphones in order to minimize input from other sounds nearby.

The domains are:

Warmth - *how warm/affectionate/kind does the culture seem?*

Competence - *how competent does the culture seem?*

Threat - *how threatening does the culture seem?*

Liking - *how much do you think you would like the culture?*

Happiness - *how happy does the culture seem?*

Evolved - *how evolved do you consider the culture to be?*

Please click the play button to listen to the song. When the song is finished, answer the following questions."

Appendix B. Linear mixed model equations for Study 1 and 2

Linear mixed model equations from Study 1:

Eq. (B.1)

Models 1-7: Equation of Musical Ratings on Musical Parameters:

$\gamma_{ij} = a + a_p + a_s + b_1 x_{1i} + b_2 x_{2i} + ... + b_5 x_{5i} + e_{ij}$

Note. γ_{ij} is the dependent variable (musical rating dimension). a is the intercept. a_p is the random intercept for participant. a_s is the random intercept for song. x_{1i} is the value of the effect of predictor 1. b_1 is the regular slope for predictor 1. e_{ij} is the remaining random variance.

Eq. (B.2)

Model 8-11: Equation of Cultural Ratings on Musical Parameters:

$\gamma_{ij} = a + a_p + a_s + b_1 x_{1i} + b_2 x_{2i} + ... + b_5 x_{5i} + e_{ij}$

Note. γ_{ij} is the dependent variable (musical rating dimension). a is the intercept. a_p is the random intercept for participant. a_s is the random intercept for song. x_{1i} is the value of the effect of predictor 1. b_1 is the regular slope for predictor 1. e_{ij} is the remaining random variance.

Linear mixed model equations from Study 2:

Eq. (B.3)

Models 16-19: Equation of Cultural Ratings on Musical Parameters:

$$\gamma_{ij} = a + a_p + a_s + (b_{p1} + b_1)x_{1i} + (b_{p2} + b_2)x_{2i} + (b_{s1} + b_1)x_{1j} + (b_{s2} + b_2)x_{2j} + b_3x_{1ij}x_{2ij} + e_{ij}$$

Note. γ_{ij} is the dependent variable (cultural evaluation dimension). a is the intercept. a_p is the random intercept for participant. a_s is the random intercept for song. b_{p1} is the slope of the random effect of predictor 1(musical parameter) by participant. x_{1i} is the value of the effect of predictor 1 by participant. b_{s1} is the slope of the random effect of predictor 1 by song. x_{1j} is the value of the effect of predictor 1 by song. b_1 is the regular slope for predictor 1. b_3 is the slope of the interaction effect between parameters. e_{ij} is the remaining random variance.

Eq. (B.4)

Models 20-25: Equation of Individual Differences on Cultural Ratings

$$\gamma_{ij} = a + a_p + a_s + (b_{s1} + b_1)x_{1j} + (b_{s2} + b_2)x_{2j} + (b_{s3} + b_3)x_{3j} + (b_{s4} + b_4)x_{4j} + (b_{p1} + b_4)x_{4i} + b_6x_{3j}x_{4ij} + b_7x_{2j}x_{3j} + e_{ij}$$

Note. γ_{ij} is the dependent variable (cultural evaluation dimension). a is the intercept. a_p is the random intercept for participant. a_s is the random intercept for song. b_{s1} is the slope of the random effect of predictor 1(gender) by song. x_{1j} is the value of the effect of predictor 1 by song. b_1 is the regular slope for predictor 1. b_4 is the regular slope for dissonance. b_6 is the slope of the interaction effect between musical training and dissonance. b_7 is the slope of the interaction effect between age and musical training. e_{ij} is the remaining random variance.

Additional Analyses:

Eq. (B.5)

Models 12-15: Equation of Cultural Ratings on Musical Ratings:

$$\gamma_{ij} = a + a_p + a_s + b_1x_{1i} + b_2x_{2i} + ... + b_7x_{7i} + e_{ij}$$

Note. γ_{ij} is the dependent variable (cultural evaluation dimension). a is the intercept. a_p is the random intercept for participant. a_s is the random intercept for song. x_{1i} is the value of the effect of predictor 1. b_1 is the regular slope for predictor 1. e_{ij} is the remaining random variance.

Eq. (B.6)

Models 26-33: Equation of Cultural Ratings on Amount of Dissonance

$$\gamma_{ij} = a + a_p + a_s + (b_{p1} + b_1)x_{1i} + e_{ij}$$

Note. γ_{ij} is the dependent variable (cultural evaluation dimension). a is the intercept. a_p is the random intercept for participant. a_s is the random intercept for song. b_{p1} is the slope of the random effect of the predictor by participant. x_{1i} is the value of the effect of the predictor by participant. b_1 is the regular slope for the predictor. e_{ij} is the remaining random variance.

Appendix C. Additional analyses

Effect of Cultural Evaluation on Musical Ratings

Supplementary Table 1

Standardized Regression Coefficients for Cultural Ratings on Musical Ratings

Variables	Warmth<br>Intercept = .000<br>*SE* = .076<br>*df* = 45.83<br>t = .000<br>*p* = .999	Threat<br>Intercept = .000<br>*SE* = .060<br>*df* = 43.43<br>t = .000<br>*p* = .999	Competence<br>Intercept = -.000<br>*SE* = .086<br>*df* = 67.63<br>t = .000<br>*p* = .999	Evolvedness<br>Intercept = .000<br>*SE* = .108<br>*df* = 56.65<br>t = .000<br>*p* = .999
Warmth	**.090(.033)<br>*p* = 0.006<br>*d* = 0.1**	**-.076(.033)<br>*p* = 0.020<br>*d* = -0.2**	-.032(.031)<br>*p* = 0.313	-.020(.026)<br>*p* = 0.453
Threat	**-.112(.027)<br>*p* < 0.001<br>*d* = -0.2**	**.227(.027)<br>*p* < 0.001<br>*d* = 0.4**	**-.105(.026)<br>*p* = 0.001<br>*d* = -0.2**	**-.065(.022)<br>*p* = 0.003<br>*d* = -0.2**
Like	.052(.029)<br>*p* = 0.077	-.036(.029)<br>*p* = 0.277	**.118(.028)<br>*p* < 0.001<br>*d* = 0.2**	**.113(.024)<br>*p* < 0.001<br>*d* = 0.2**
Happiness	**.097(.036)<br>*p* = 0.008<br>*d* = 0.2**	-.013(.036)<br>*p* = 0.392	.034(.035)<br>*p* = 0.323	**.101(.029)<br>*p* = 0.005<br>*d* = 0.2**
Energy	.050(.033)<br>*p* = 0.132	.044(.033)<br>*p* = 0.184	**.067(.032)<br>*p* = 0.035<br>*d* = 0.2**	.116(.026)<br>*p* = 0.549
Advanced	.050(.026)<br>*p* = 0.056	-.018(.026)<br>*p* = 0.496	**.141(.025)<br>*p* < 0.001<br>*d* = 0.2**	**.060(.021)<br>*p* = 0.006<br>*d* = 0.2**
Familiarity	-.022(.027)<br>*p* = 0.412	.037(.028)<br>*p* = 0.191	.043(.027)<br>*p* = 0.113	.017(.023)<br>*p* = 0.459

Note. Standard Deviations in parentheses. Significance below .05 in BOLD

Discussion

The results indicated quite a consistent effect of musical perceptions on cultural evaluations. The most notable of these predictors was the musical threat rating that moderately positively predicted cultural threat, which is not surprising given that they represent corresponding dimensions. The results also indicate that the evaluation of cultural warmth is not predominantly dependent on the evaluation of music in the same domain, as both musical happiness and musical energy, compared to musical warmth, appeared to be stronger predictors of cultural warmth. Additionally, the positive effect of musical happiness on cultural warmth, and the positive effect of musical liking on cultural competence further

illustrate the independence of the cultural warmth and cultural competence dimensions. The fact that liking predicts competence may not be surprising, considering that the competence dimension reflects ability, popularity and higher social status - characteristics which are generally admired by others.

Effect of Cultural Evaluation on amount of dissonance

Supplementary Table 2

Standardized Regression Coefficients for Cultural Ratings on the Amount of dissonance

Variables	Warmth<br>Intercept = -.174<br>SE = .152<br>df = 4.77<br>t-value = -1.14<br>*p* = .308	Threat<br>Intercept = -.139<br>SE = .154<br>df = 4.92<br>t-value = .905<br>*p* = .408	Competence<br>Intercept = .038<br>SE = .135<br>df = 4.73<br>t-value = .248<br>*p* = .792	Evolved<br>Intercept = .004<br>SE = .113<br>df = 7.02<br>t-value = .038<br>*p* = .971
Percentage of dissonant intervals	-.136(.126)<br>*p* = 0.342	.092(.126)<br>*p* = 0.508	-.122(.113)<br>*p* = 0.339	-.083(.085)<br>*p* = 0.386
Variables	Warmth<br>Intercept = -.146<br>SE = .289<br>df = 4.26<br>t-value = -.505<br>*p* = .639	Threat<br>Intercept = -.077<br>SE = .240<br>df = 4.24<br>t-value = -.321<br>*p* = .763	Competence<br>Intercept = -.229<br>SE = .257<br>df = 4.35<br>t-value = -.893<br>*p* = .419	Evolved<br>Intercept = -.108<br>SE = .204<br>df = 5.45<br>t-value = -.531<br>*p* = .616
Percentage of transposed tones	-.049(.279)<br>*p* = 0.261	.308(.230)<br>*p* = 0.253	.177(.246)<br>*p* = 0.512	.047(.188)<br>*p* = 0.813

Note. Categorical predictors are contrast coded. Standard Deviations in parentheses.

Discussion:

Since dissonance vs. consonance was only included as a dichotomous variable in the original models, an additional eight models were created in order to investigate whether the amount of dissonance present in the dissonant melodies could predict any of the cultural evaluations. Four models tested the percentage of dissonant intervals created, and four models tested the percentage of tones moved. There were no effects of the number of dissonant intervals or the number of altered tones on any of the cultural evaluations. This could be due to participants creating a general binary impression of the culture by the mere presence of dissonance in the melody. Such a processing style may be less sensitive to the specific degree of dissonance throughout the melody. On the other hand, it could be that the variation in the degree of dissonance within the dissonant melodies was too limited in order to elicit any significant differences in cultural evaluations.
